# Redetermination of (2,2′-bipyridine-κ^2^ *N*,*N*′)dichlorido­palladium(II) dichloro­methane solvate

**DOI:** 10.1107/S1600536809016262

**Published:** 2009-05-07

**Authors:** Nam-Ho Kim, In-Chul Hwang, Kwang Ha

**Affiliations:** a School of Applied Chemical Engineering, The Research Institute of Catalysis, Chonnam National University, Gwangju 500-757, Republic of Korea; b Institute of Basic Sciences, Pohang University of Science and Technology, Pohang 790-784, Republic of Korea

## Abstract

In the title compound, [PdCl_2_(C_10_H_8_N_2_)]·CH_2_Cl_2_, the Pd^2+^ ion is four-coordinated in a slightly distorted square-planar environment by two N atoms of the 2,2′-bipyridine (bipy) ligand and two chloride ions. The compound displays intra­molecular C—H⋯Cl hydrogen bonds and pairs of complex mol­ecules are connected by inter­molecular C—H⋯Cl hydrogen bonds. Inter­molecular π–π inter­actions are present between the pyridine rings of the ligand, the shortest centroid–centroid distance being 4.096 (3) Å. As a result of the electronic nature of the chelate ring, it is possible to create π–π inter­actions to its symmetry-related counterpart [3.720 (2) Å] and also with a pyridine ring [3.570 (3) Å] of the bipy unit. The present structure is a redetermination of a previous structure [Vicente *et al.* (1997 ▶). Private communication (refcode PYCXMN02). CCDC, Cambridge, England]. In the new structure refinement all H atoms were located in a difference Fourier synthesis. Their coordinates were refined freely, together with isotropic displacement parameters.

## Related literature

For crystal structures of [Pd*X*_2_(bipy)] (*X* = Cl or Br), see: Maekawa *et al.* (1991 ▶); Vicente *et al.* (1997 ▶); Smeets *et al.* (1997 ▶). For related Pt(II, IV)–bipyridine complexes, see: Osborn & Rogers (1974 ▶); Hambley (1986 ▶); Sartori *et al.* (2005 ▶); Momeni *et al.* (2007 ▶); Kim *et al.* (2009 ▶).
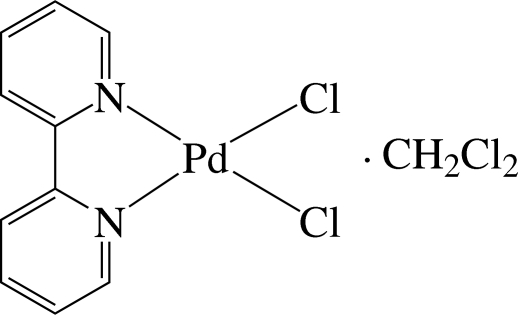


## Experimental

### 

#### Crystal data

[PdCl_2_(C_10_H_8_N_2_)]·CH_2_Cl_2_
*M* *_r_* = 418.41
Triclinic, 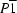

*a* = 8.7913 (10) Å
*b* = 9.1115 (11) Å
*c* = 10.1846 (12) Å
α = 72.481 (2)°
β = 66.983 (2)°
γ = 81.429 (2)°
*V* = 715.58 (15) Å^3^
*Z* = 2
Mo *K*α radiation
μ = 2.03 mm^−1^
*T* = 293 K
0.20 × 0.08 × 0.08 mm

#### Data collection

Bruker SMART 1000 CCD diffractometer
Absorption correction: multi-scan (*SADABS*; Bruker, 2000 ▶) *T*_min_ = 0.623, *T*_max_ = 0.850
4221 measured reflections
2862 independent reflections
2298 reflections with *I* > 2σ(*I*)
*R*_int_ = 0.023

#### Refinement

*R*[*F* ^2^ > 2σ(*F* ^2^)] = 0.044
*wR*(*F* ^2^) = 0.097
*S* = 1.05
2862 reflections
203 parameters
All H-atom parameters refined
Δρ_max_ = 0.56 e Å^−3^
Δρ_min_ = −0.69 e Å^−3^

### 

Data collection: *SMART* (Bruker, 2000 ▶); cell refinement: *SAINT* (Bruker, 2000 ▶); data reduction: *SAINT*; program(s) used to solve structure: *SHELXS97* (Sheldrick, 2008 ▶); program(s) used to refine structure: *SHELXL97* (Sheldrick, 2008 ▶); molecular graphics: *ORTEP-3* (Farrugia, 1997 ▶) and *PLATON* (Spek, 2009 ▶); software used to prepare material for publication: *SHELXL97*.

## Supplementary Material

Crystal structure: contains datablocks global, I. DOI: 10.1107/S1600536809016262/kp2220sup1.cif

Structure factors: contains datablocks I. DOI: 10.1107/S1600536809016262/kp2220Isup2.hkl

Additional supplementary materials:  crystallographic information; 3D view; checkCIF report

## Figures and Tables

**Table 1:** Selected bond lengths (Å)

Pd1—N2	2.025 (4)
Pd1—N1	2.029 (4)
Pd1—Cl2	2.2853 (14)
Pd1—Cl1	2.2964 (14)

**Table 2:** Hydrogen-bond geometry (Å, °)

*D*—H⋯*A*	*D*—H	H⋯*A*	*D*⋯*A*	*D*—H⋯*A*
C1—H1⋯Cl2	0.93 (5)	2.59 (5)	3.230 (6)	126 (4)
C2—H2⋯Cl2^i^	0.93 (5)	2.79 (5)	3.595 (7)	145 (4)
C10—H10⋯Cl1	0.86 (5)	2.68 (5)	3.248 (6)	125 (4)
C11—H11*B*⋯Cl1	0.99 (6)	2.63 (6)	3.578 (8)	161 (5)

Symmetry code: (i) 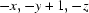.

**Fig. 1.**
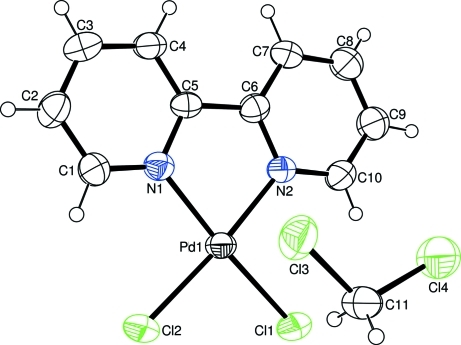
The structure of the title compound, with displacement ellipsoids drawn at the 40% probability level for non-H atoms.

**Fig. 2.**
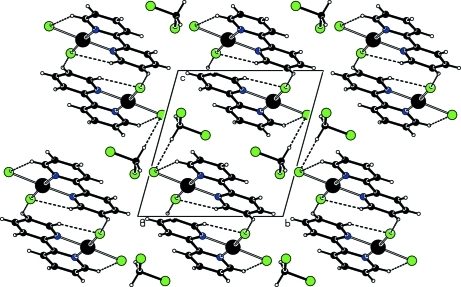
Crystal packing of I. Hydrogen-bond interactions are drawn with dashed lines.

## supplementary crystallographic information

### Comment

The asymmetric unit of the title compound, [PdCl_2_(C_10_H_8_N_2_)].CH_2_Cl_2_, contains a neutral Pd^II^ complex and a solvent molecule (Fig. 1). The compound crystallized in the triclinic space group *P*1, whereas the previously reported complex [PdCl_2_(C_10_H_8_N_2_)] crystallized in the orthorhombic space group *C*222_1_ (Maekawa *et al.*, 1991). The X-ray structure analysis of the title compound was previously carried out (Vicente *et al.*, 1997) and the structure is stored in CCDC code: RAGVUQ. The stored and our crystal structures are in agreement, however, the new structure determination provides data on hydrogen bonding based on H atoms positions located from a difference Fourier synthesis and refined isotropically.

In the complex, the Pd^2+^ ion is four-coordinated in a distorted square-planar environment by two N atoms of the 2,2'-bipyridine (bipy) ligand and two Cl ions. The main contribution to the distortion is the tight N1—Pd1—N2 chelate angle (81.04 (16)°), which results in non-linear *trans* arrangement (<N1—Pd1—Cl1 = 175.78 (12)° and <N2—Pd1—Cl2 = 174.97 (12)°). The Pd1—N and Pd1—Cl bond lengths are almost equal (Pd1—N: 2.029 (4) and 2.025 (4) Å; Pd1—Cl 2.2964 (14) and 2.2853 (14) Å), respectively (Table 1), and close to those reported for [PdCl_2_(C_10_H_8_N_2_)] (Maekawa *et al.*, 1991). The best least-squares plane of the atoms of the planar complex, except Cl2, reveals mean deviation of 0.026 (5) Å; deviation of Cl2 from this plane is 0.184 (2) Å. The compound displays the inter- and intramolecular C—H···Cl hydrogen bonds (Table 2) and molecules are connected by intermolecular hydrogen bonds and stacked in layres along the *c* axis (Fig. 2). Intermolecular π-π interactions (defined by separation distance between the ring centroids (the symmetry operation for second plane 1-x,1-y,-z) involve the planes: Pd1,N1→ N2 and ity symmetry related counterpart, 3.720 (2) Å, planes are parallel and shifted for 1.506 Å; Pd1,N1→ N2 and N1→ C5, 3.570 (3) Å, the dihedral angle between planes is 1.54 °, no shift; N1→ C5 and N2→ C10, 4.096 (3) Å the dihedral angle between planes is 2.72 °, no shift.

### Experimental

To a solution of Na_2_PdCl_4_ (0.300 g, 1.020 mmol) in EtOH (30 ml) was added 2,2'-bipyridine (0.159 g, 1.018 mmol) and stirred for 5 h at room temperature. The precipitate obtained was separated by filtration and washed with EtOH and water and dried under vacuum, to give a yellow powder (0.302 g). Crystals suitable for X-ray analysis were obtained by slow evaporation from a CH_2_Cl_2_ solution.

### Refinement

All H atoms were located from Fourier difference maps and refined isotropically.

### Crystal data

**Table:**

[PdCl_2_(C_10_H_8_N_2_)]·CH_2_Cl_2_	*Z* = 2
*M**_r_* = 418.41	*F*(000) = 408
Triclinic, *P*1	*D*_x_ = 1.942 Mg m^−^^3^
Hall symbol: -P 1	Mo *K*α radiation, λ = 0.71073 Å
*a* = 8.7913 (10) Å	Cell parameters from 983 reflections
*b* = 9.1115 (11) Å	θ = 2.3–21.7°
*c* = 10.1846 (12) Å	µ = 2.03 mm^−^^1^
α = 72.481 (2)°	*T* = 293 K
β = 66.983 (2)°	Stick, yellow
γ = 81.429 (2)°	0.20 × 0.08 × 0.08 mm
*V* = 715.58 (15) Å^3^	

### Data collection

**Table:**

Bruker SMART 1000 CCD diffractometer	2862 independent reflections
Radiation source: fine-focus sealed tube	2298 reflections with *I* > 2σ(*I*)
graphite	*R*_int_ = 0.023
φ and ω scans	θ_max_ = 26.4°, θ_min_ = 2.3°
Absorption correction: multi-scan (*SADABS*; Bruker, 2000)	*h* = −10→10
*T*_min_ = 0.623, *T*_max_ = 0.850	*k* = −7→11
4221 measured reflections	*l* = −12→12

### Refinement

**Table:**

Refinement on *F*^2^	Primary atom site location: structure-invariant direct methods
Least-squares matrix: full	Secondary atom site location: difference Fourier map
*R*[*F*^2^ > 2σ(*F*^2^)] = 0.044	Hydrogen site location: inferred from neighbouring sites
*wR*(*F*^2^) = 0.097	All H-atom parameters refined
*S* = 1.05	*w* = 1/[σ^2^(*F*_o_^2^) + (0.0352*P*)^2^ + 0.5604*P*] where *P* = (*F*_o_^2^ + 2*F*_c_^2^)/3
2862 reflections	(Δ/σ)_max_ < 0.001
203 parameters	Δρ_max_ = 0.56 e Å^−^^3^
0 restraints	Δρ_min_ = −0.69 e Å^−^^3^

### Special details

**Table:**

Geometry. All e.s.d.'s (except the e.s.d. in the dihedral angle between two l.s. planes) are estimated using the full covariance matrix. The cell e.s.d.'s are taken into account individually in the estimation of e.s.d.'s in distances, angles and torsion angles; correlations between e.s.d.'s in cell parameters are only used when they are defined by crystal symmetry. An approximate (isotropic) treatment of cell e.s.d.'s is used for estimating e.s.d.'s involving l.s. planes.
Refinement. Refinement of *F*^2^ against ALL reflections. The weighted *R*-factor *wR* and goodness of fit *S* are based on *F*^2^, conventional *R*-factors *R* are based on *F*, with *F* set to zero for negative *F*^2^. The threshold expression of *F*^2^ > σ(*F*^2^) is used only for calculating *R*-factors(gt) *etc*. and is not relevant to the choice of reflections for refinement. *R*-factors based on *F*^2^ are statistically about twice as large as those based on *F*, and *R*- factors based on ALL data will be even larger.

### Fractional atomic coordinates and isotropic or equivalent isotropic displacement parameters (Å^2^)

**Table:**

	*x*	*y*	*z*	*U*_iso_*/*U*_eq_	
Pd1	0.32112 (5)	0.28572 (4)	0.21130 (4)	0.04479 (15)	
Cl1	0.36850 (18)	0.02764 (15)	0.30403 (16)	0.0612 (4)	
Cl2	0.1458 (2)	0.22805 (17)	0.11857 (19)	0.0720 (5)	
N1	0.2916 (5)	0.5169 (5)	0.1348 (4)	0.0454 (10)	
N2	0.4786 (5)	0.3551 (5)	0.2803 (4)	0.0446 (10)	
C1	0.1884 (7)	0.5895 (7)	0.0665 (6)	0.0545 (14)	
H1	0.126 (6)	0.526 (6)	0.052 (5)	0.058 (16)*	
C2	0.1803 (8)	0.7475 (7)	0.0175 (7)	0.0610 (15)	
H2	0.101 (7)	0.800 (6)	−0.020 (6)	0.063 (17)*	
C3	0.2768 (8)	0.8325 (7)	0.0413 (7)	0.0620 (16)	
H3	0.273 (6)	0.930 (6)	0.018 (5)	0.043 (14)*	
C4	0.3831 (7)	0.7585 (6)	0.1120 (6)	0.0515 (13)	
H4	0.451 (6)	0.809 (6)	0.128 (5)	0.046 (14)*	
C5	0.3887 (6)	0.6010 (6)	0.1590 (5)	0.0417 (11)	
C6	0.4959 (6)	0.5099 (6)	0.2384 (5)	0.0466 (12)	
C7	0.6069 (8)	0.5739 (7)	0.2675 (7)	0.0644 (16)	
H7	0.623 (7)	0.683 (7)	0.236 (6)	0.081 (19)*	
C8	0.7003 (9)	0.4784 (8)	0.3427 (8)	0.077 (2)	
H8	0.775 (8)	0.512 (8)	0.355 (7)	0.09 (2)*	
C9	0.6833 (8)	0.3223 (8)	0.3858 (8)	0.0717 (18)	
H9	0.734 (6)	0.255 (6)	0.442 (5)	0.049 (15)*	
C10	0.5692 (7)	0.2645 (7)	0.3546 (7)	0.0579 (15)	
H10	0.562 (6)	0.166 (6)	0.376 (5)	0.043 (14)*	
C11	0.0507 (9)	0.1175 (7)	0.6220 (8)	0.0614 (16)	
H11A	−0.043 (7)	0.071 (6)	0.685 (6)	0.062 (17)*	
H11B	0.115 (7)	0.087 (7)	0.529 (7)	0.09 (2)*	
Cl3	−0.0049 (2)	0.31218 (19)	0.55636 (18)	0.0740 (5)	
Cl4	0.1895 (2)	0.1016 (2)	0.7099 (2)	0.0887 (6)	

### Atomic displacement parameters (Å^2^)

**Table:**

	*U*^11^	*U*^22^	*U*^33^	*U*^12^	*U*^13^	*U*^23^
Pd1	0.0465 (2)	0.0387 (2)	0.0512 (3)	−0.00670 (17)	−0.01825 (18)	−0.01196 (18)
Cl1	0.0744 (9)	0.0386 (7)	0.0735 (9)	−0.0059 (7)	−0.0309 (8)	−0.0121 (7)
Cl2	0.0836 (11)	0.0561 (9)	0.0965 (12)	−0.0203 (8)	−0.0543 (9)	−0.0117 (8)
N1	0.045 (2)	0.044 (2)	0.048 (2)	−0.005 (2)	−0.017 (2)	−0.0118 (19)
N2	0.049 (2)	0.039 (2)	0.050 (2)	−0.0036 (19)	−0.021 (2)	−0.0121 (19)
C1	0.052 (3)	0.054 (3)	0.062 (3)	−0.002 (3)	−0.028 (3)	−0.012 (3)
C2	0.063 (4)	0.050 (4)	0.068 (4)	0.008 (3)	−0.029 (3)	−0.009 (3)
C3	0.072 (4)	0.039 (3)	0.074 (4)	−0.002 (3)	−0.025 (3)	−0.016 (3)
C4	0.057 (3)	0.039 (3)	0.063 (3)	−0.005 (3)	−0.025 (3)	−0.016 (3)
C5	0.041 (3)	0.039 (3)	0.044 (3)	−0.004 (2)	−0.010 (2)	−0.016 (2)
C6	0.046 (3)	0.044 (3)	0.045 (3)	−0.004 (2)	−0.012 (2)	−0.011 (2)
C7	0.080 (4)	0.052 (4)	0.078 (4)	−0.015 (3)	−0.045 (4)	−0.013 (3)
C8	0.084 (5)	0.070 (5)	0.097 (5)	−0.016 (4)	−0.058 (4)	−0.009 (4)
C9	0.074 (4)	0.063 (4)	0.092 (5)	−0.004 (3)	−0.054 (4)	−0.009 (4)
C10	0.065 (4)	0.045 (3)	0.067 (4)	−0.002 (3)	−0.032 (3)	−0.008 (3)
C11	0.065 (4)	0.052 (4)	0.072 (4)	−0.009 (3)	−0.026 (3)	−0.019 (3)
Cl3	0.0761 (10)	0.0652 (10)	0.0759 (10)	0.0022 (8)	−0.0354 (9)	−0.0044 (8)
Cl4	0.0906 (12)	0.0701 (11)	0.1211 (15)	0.0040 (9)	−0.0663 (12)	−0.0139 (10)

### Geometric parameters (Å, °)

**Table:**

Pd1—N2	2.025 (4)	C4—H4	0.90 (5)
Pd1—N1	2.029 (4)	C5—C6	1.480 (7)
Pd1—Cl2	2.2853 (14)	C6—C7	1.371 (7)
Pd1—Cl1	2.2964 (14)	C7—C8	1.377 (9)
N1—C1	1.335 (6)	C7—H7	0.96 (6)
N1—C5	1.360 (6)	C8—C9	1.369 (9)
N2—C10	1.338 (7)	C8—H8	0.83 (6)
N2—C6	1.359 (6)	C9—C10	1.375 (8)
C1—C2	1.375 (8)	C9—H9	0.90 (5)
C1—H1	0.93 (5)	C10—H10	0.86 (5)
C2—C3	1.360 (8)	C11—Cl4	1.743 (6)
C2—H2	0.93 (5)	C11—Cl3	1.765 (6)
C3—C4	1.376 (8)	C11—H11A	0.90 (5)
C3—H3	0.85 (5)	C11—H11B	0.99 (6)
C4—C5	1.369 (7)		
			
N2—Pd1—N1	81.04 (16)	N1—C5—C4	120.6 (5)
N2—Pd1—Cl2	174.97 (12)	N1—C5—C6	115.2 (4)
N1—Pd1—Cl2	94.27 (12)	C4—C5—C6	124.2 (5)
N2—Pd1—Cl1	94.74 (12)	N2—C6—C7	121.4 (5)
N1—Pd1—Cl1	175.78 (12)	N2—C6—C5	115.0 (4)
Cl2—Pd1—Cl1	89.94 (5)	C7—C6—C5	123.6 (5)
C1—N1—C5	119.3 (5)	C6—C7—C8	118.8 (6)
C1—N1—Pd1	126.5 (4)	C6—C7—H7	122 (4)
C5—N1—Pd1	114.2 (3)	C8—C7—H7	119 (4)
C10—N2—C6	118.9 (4)	C9—C8—C7	120.2 (6)
C10—N2—Pd1	126.6 (4)	C9—C8—H8	118 (5)
C6—N2—Pd1	114.4 (3)	C7—C8—H8	121 (5)
N1—C1—C2	121.4 (6)	C8—C9—C10	118.5 (6)
N1—C1—H1	116 (3)	C8—C9—H9	124 (3)
C2—C1—H1	123 (3)	C10—C9—H9	117 (3)
C3—C2—C1	119.7 (6)	N2—C10—C9	122.2 (6)
C3—C2—H2	118 (4)	N2—C10—H10	118 (3)
C1—C2—H2	122 (4)	C9—C10—H10	119 (3)
C2—C3—C4	119.2 (6)	Cl4—C11—Cl3	111.0 (3)
C2—C3—H3	124 (3)	Cl4—C11—H11A	111 (4)
C4—C3—H3	117 (3)	Cl3—C11—H11A	106 (4)
C5—C4—C3	119.8 (5)	Cl4—C11—H11B	106 (3)
C5—C4—H4	118 (3)	Cl3—C11—H11B	103 (4)
C3—C4—H4	123 (3)	H11A—C11—H11B	120 (5)
			
N2—Pd1—N1—C1	−177.3 (4)	C3—C4—C5—N1	0.8 (8)
Cl2—Pd1—N1—C1	4.6 (4)	C3—C4—C5—C6	−178.8 (5)
N2—Pd1—N1—C5	2.4 (3)	C10—N2—C6—C7	1.7 (8)
Cl2—Pd1—N1—C5	−175.7 (3)	Pd1—N2—C6—C7	−175.3 (4)
N1—Pd1—N2—C10	179.8 (5)	C10—N2—C6—C5	−179.1 (4)
Cl1—Pd1—N2—C10	−0.3 (5)	Pd1—N2—C6—C5	4.0 (5)
N1—Pd1—N2—C6	−3.5 (3)	N1—C5—C6—N2	−2.0 (6)
Cl1—Pd1—N2—C6	176.4 (3)	C4—C5—C6—N2	177.6 (5)
C5—N1—C1—C2	1.2 (8)	N1—C5—C6—C7	177.3 (5)
Pd1—N1—C1—C2	−179.2 (4)	C4—C5—C6—C7	−3.1 (8)
N1—C1—C2—C3	−1.3 (9)	N2—C6—C7—C8	−1.0 (9)
C1—C2—C3—C4	1.1 (9)	C5—C6—C7—C8	179.8 (6)
C2—C3—C4—C5	−0.8 (9)	C6—C7—C8—C9	0.7 (11)
C1—N1—C5—C4	−0.9 (7)	C7—C8—C9—C10	−1.2 (11)
Pd1—N1—C5—C4	179.4 (4)	C6—N2—C10—C9	−2.2 (9)
C1—N1—C5—C6	178.7 (4)	Pd1—N2—C10—C9	174.4 (5)
Pd1—N1—C5—C6	−1.0 (5)	C8—C9—C10—N2	1.9 (10)

### Hydrogen-bond geometry (Å, °)

**Table:**

*D*—H···*A*	*D*—H	H···*A*	*D*···*A*	*D*—H···*A*
C1—H1···Cl2	0.93 (5)	2.59 (5)	3.230 (6)	126 (4)
C2—H2···Cl2^i^	0.93 (5)	2.79 (5)	3.595 (7)	145 (4)
C10—H10···Cl1	0.86 (5)	2.68 (5)	3.248 (6)	125 (4)
C11—H11B···Cl1	0.99 (6)	2.63 (6)	3.578 (8)	161 (5)

Symmetry codes: (i) −*x*, −*y*+1, −*z*.
